# Traditional, Complementary, and Alternative Medicine for Self-Care in Chronic Neck and Shoulder Pain: A Scoping Review

**DOI:** 10.3390/nursrep16020044

**Published:** 2026-01-29

**Authors:** Thi Khanh Ha Doan, Ernesta Sofija, Thu-Hang Ho-Thi, Nguyen Ngoc Phan, Hiep N. Le, Lan N. C. Nguyen, Hai Phung

**Affiliations:** 1 School of Medicine and Dentistry, Griffith University, Gold Coast, QLD 4222, Australia; thikhanhha.doan@griffithuni.edu.au (T.K.H.D.); e.sofija@griffith.edu.au (E.S.); t.hangthi@griffith.edu.au (T.-H.H.-T.); nguyenngoc.phan@griffithuni.edu.au (N.N.P.); ngochiep.le@griffithuni.edu.au (H.N.L.); 2 Faculty of Health Sciences, Cuu Long University, Vinh Long 890000, Vietnam; 3 Faculty of Traditional Medicine, Can Tho University of Medicine and Pharmacy, Can Tho 90000, Vietnam; nnclan@ctump.edu.vn; 4 Vinh Long Health Department, Vinh Long 890000, Vietnam

**Keywords:** neck pain, shoulder pain, traditional medicine, complementary therapies, alternative medicine, integrative medicine, self-care

## Abstract

**Background:** Traditional, complementary, and alternative medicine (TCAM) is a promising and increasingly popular approach in managing chronic neck and shoulder pain. Despite recognized benefits for pain relief and well-being, how TCAM facilitates self-care practices is poorly understood. This scoping review maps the existing evidence on TCAM-based self-care strategies for adults with chronic neck and shoulder pain to identify which domains of self-care are addressed and which are overlooked. **Methods:** We searched five academic databases, including PubMed, Scopus, Embase, Cinahl Complete (EBSCOhost), and Public Health Database (ProQuest). Articles published over a 20-year period that examined the use of TCAM for adults with chronic neck and shoulder pain were included. An analytical framework based on Riegel’s three core components of self-care (maintenance, monitoring, and management) was used to structure and synthesize the findings from included studies. **Results:** Thirty-three studies met the inclusion criteria. Most research focused on just one or two self-care components, primarily self-care maintenance (e.g., physical activities and treatment adherence) and self-care management (e.g., pain control). However, critical dimensions, such as psychological well-being, social support, work–life balance, and cultural context, were frequently overlooked. **Conclusions:** To enhance the quality of life for adults with chronic neck and shoulder pain, future TCAM research must adopt a more holistic self-care approach that extends beyond physical symptoms. Our findings highlight the need for integrated research, policy, and clinical services that address the full spectrum of self-care in chronic pain management.

## 1. Introduction

Complementary and alternative medicine (CAM) encompasses a diverse group of medical systems, practices, and products not traditionally considered part of conventional medicine and is not fully integrated into the mainstream healthcare system [1,2]. These practices, sometimes referred to simply as “traditional medicine” [3], can often be categorized into five major domains: (1) whole medical systems (e.g., naturopathy and traditional Chinese medicine); (2) mind–body medicine (e.g., yoga and meditation); (3) biologically based practices (e.g., herbal medicine and diet therapies); (4) manipulative and body-based practices (e.g., exercise and massage); and (5) energy medicine (e.g., acupuncture, reiki, Qi gong, and Tai Chi) [1]. Collectively, TCAM is associated with a range of benefits, including improvements in metabolic health, reductions in stress, anxiety, and depression, and potential healthcare cost savings [4,5,6,7].

For pain management, TCAM offers several effective strategies, including acupuncture, mind–body techniques, cupping, and massage therapy [8,9,10,11]. The prevalence of this issue is significant; in the United States, more than 1 in 5 adults report experiencing pain daily or on most days and experience impacts on their daily and social activities [12]. Furthermore, chronic pain is associated with higher rates of depression and reduced physical activity, diminishing quality of life, and contributing to a significant societal burden [13,14]. The economic impact is also substantial, with chronic pain costing an estimated USD 560 billion annually from lost productivity, direct medical costs, and disability programs [15].

Despite this, in routine clinical practice, healthcare professionals often focus narrowly on the painful condition itself. As a result, patient self-management strategies are frequently ignored, overlooked during assessment, and left unincorporated into care plans. The World Health Organization (2005) emphasized the importance of self-managing chronic conditions at both the individual and population levels [16]. Therefore, developing policies and clinical practices that systematically evaluate and integrate these self-management strategies is crucial for helping patients alleviate pain during and after hospitalization.

Musculoskeletal disorders, particularly spine-related pain and disability, are recognized as a significant global threat to healthy aging [17]. Following the COVID-19 pandemic, a higher incidence of musculoskeletal pain has been reported [18,19]. Consequently, the trend in the use of TCAM among people suffering from chronic neck and shoulder pain has shown a notable rise. Therefore, cervical pain is a prevalent health issue affecting people of all ages, not just the aging population, in modern societies [20,21].

Another motivation for using TCAM during episodes of neck pain includes skepticism toward conventional medicine (28%) and the financial burden of out-of-pocket expenses (13%) [22]. Despite current policies encouraging those with chronic neck and shoulder pain to seek conventional medical care, many individuals still have confidence in TCAM. For example, adults who self-manage their spinal health through physical activity can enhance muscle strength, reduce the risk of chronic diseases, alleviate anxiety and stress, and help maintain meaningful social connections [23]. Clinical guidelines also advocate for addressing psychological factors and adopting active self-management strategies, including relaxation techniques, exercise, and mindfulness [24]. Given these trends, there is a pressing need for research that systematically maps TCAM-based self-care strategies for managing chronic neck and shoulder pain. Understanding the impact of these approaches is essential for improving outcomes not only for individuals but also for their families and communities.

Self-management for spine pain requires behavior changes and clear communication between practitioners and their patients [25]. The reason for this is that mid-life and older adults have unique motivations for maintaining musculoskeletal health, including accessing social engagement, fear of falling, and exercising for fitness and to feel young [26,27]. Additionally, safety is the most important aspect of self-management strategies. Therefore, healthcare professionals and their patients must work as a team to obtain enough knowledge and skills so that the patients can have confidence to practice by themselves [28]. Furthermore, multimodal training has demonstrated beneficial effects on both functional outcomes and symptom relief in individuals with chronic neck and shoulder pain [29]. Consequently, Riegel’s theory is applicable not only to patients or nurses but also to all healthcare professionals involved in promoting self-care for people with chronic neck and shoulder pain.

**Figure 1:**
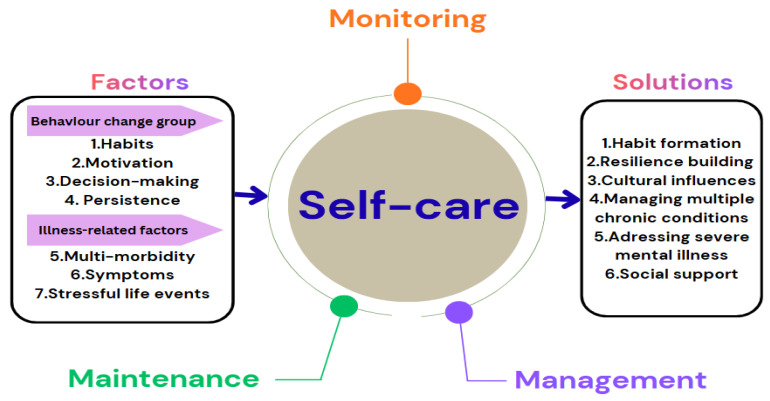
Self-care components adopted from a middle-range theory of self-care of chronic illness [30,31,32].

This scoping review uses Riegel’s (2012) middle-range theory of self-care of chronic illness (Figure 1), which defines self-care as maintaining health through proactive behavior and effective illness management [30]. The theory comprises three key components: (i) self-care maintenance—daily health-promoting practices like sleep, exercise, nutrition, and treatment adherence; (ii) self-care monitoring—tracking health indicators including physical symptoms (pain, stress, or temperature), sensations, daily routines, or cognitive processes; and (iii) self-care management—responding to symptoms through medication, activity adjustments, or seeking support. In 2018, this theory was refined to clarify the link between symptoms and self-care [31], yet the complexity of self-care behaviors remains poorly understood. Riegel (2021) expanded on barriers to self-care and highlighted key knowledge gaps, identifying seven influencing factors grouped into two categories: behavioral (habits, motivation, decision-making, and persistence) and illness-related (multimorbidity, symptoms, and stressful life events) [32]. Six proposed solutions include habit formation, resilience-building, cultural influences, managing multiple chronic conditions, addressing severe mental illness, and social support. This framework offers a comprehensive view of self-care components, highlighting both addressed areas and existing gaps.

This study is important for four key reasons. First, it explores the self-care strategies employed by adults with chronic neck and shoulder pain within TCAM practices, offering insights into how individuals manage pain in everyday life. Second, it identifies the most frequently associated self-care components within TCAM, helping to clarify which practices are commonly integrated into pain management routines. Third, it examines the factors influencing self-care behaviors during pain episodes, including personal, cultural, and contextual elements. Finally, it highlights recommended solutions to enhance self-care during pain episodes, contributing to more effective and sustainable approaches for individuals, healthcare providers, and policymakers. To achieve these aims, this scoping review will systematically investigate the multifaceted nature of TCAM-related self-care strategies for managing chronic neck and shoulder pain, addressing gaps in the existing literature and guiding future interventions.

In this review, the following research questions are addressed:

What self-care strategies are employed by adults with chronic neck and shoulder pain within TCAM practices?

Which self-care components are most frequently associated with TCAM in pain management?

What factors are related to self-care practices during pain episodes?

What solutions are recommended to enhance self-care during pain episodes?

## 2. Materials and Methods

### 2.1. Design

This scoping review was conducted in accordance with the Preferred Reporting Items for Systematic Reviews and Meta-Analyses-Extension for Scoping Reviews (PRISMA-ScR) guidelines [33] and the Joanna Briggs Institute (JBI) Manual for Evidence Synthesis [34]. The protocol was registered on Figshare (DOI: 10.6084/m9.figshare.28955390). A completed PRISMA-ScR checklist is provided in the Supplementary Materials.

### 2.2. Eligibility Criteria

The inclusion criteria were guided by the PICO framework [35]:

- Population: Adults (≥18 years) with chronic neck and/or shoulder pain.
- Intervention: Any TCAM-based self-care strategy (excluding pharmacological interventions). This also includes integrative approaches combining TCAM with conventional care.
- Outcomes: Pain, disability, psychosocial variables, or other relevant outcomes.
- Study Types: Original peer-reviewed research articles published between 2004 and November 2024, with the timeframe selected due to the increased utilization of CAM since 2010 and its association with multi-level self-care approaches since 2005 [16,36].

In this review, disability was operationalized using validated self-report instruments widely used in musculoskeletal research, including the Neck Disability Index (NDI), Northwick Park Neck Pain Questionnaire (NPQ), Copenhagen Neck Functional Disability Scale, and similar tools. These measures assess functional limitations related to neck and shoulder pain rather than formal diagnostic criteria such as those defined by the Americans with Disabilities Act (ADA).

Language: Initially, only English-language studies were included. Following reviewer feedback, we have expanded eligibility to include studies published in other languages if they provide an English abstract and sufficient methodological detail for data extraction. This change did not require a full re-execution of the search; instead, previously excluded records were rescreened under the revised criterion. Ultimately, all included studies were either published in English or had comprehensive English abstracts, and no full-text non-English studies were incorporated. This limitation is noted in the Section 4.

Exclusions: Systematic reviews, conference abstracts, letters, opinions, books, and protocols were excluded. No geographic restrictions were applied.

### 2.3. Search Strategy and Information Sources

A comprehensive literature search was conducted in five databases: PubMed, Scopus, Embase, CINAHL Complete (EBSCOhost), and Public Health Database (ProQuest). The search strategy combined terms for neck/shoulder pain, TCAM interventions, and self-care concepts. The full search strategy for each database is provided in Appendix A. Reference lists of included studies were hand-searched for additional articles.

### 2.4. Study Selection

All records were imported into EndNote version 21 and screened in Covidence (https://app.covidence.org/reviews/active, accessed on 5 November 2024). Two independent reviewers (T.K.H.D. and N.N.P.) screened titles and abstracts against eligibility criteria. Full texts of potentially relevant studies were assessed independently. Discrepancies were resolved through discussion or consultation with a third reviewer.

### 2.5. Data Charting Process

Data charting followed an iterative process recommended by JBI:

- A data extraction template was developed and piloted on five studies, then refined for clarity and completeness.
- Extracted variables included author(s), year, country, study design, setting, sample characteristics, intervention details, outcomes, and key findings.
- Additional columns captured self-care components (maintenance, monitoring, and management) and behaviors based on Riegel’s middle-range theory of self-care of chronic illness.
- Two reviewers independently charted data; discrepancies were resolved through consensus.

The final charting table is presented in Appendix B and Appendix C.

### 2.6. Synthesis of Results

Findings were synthesized using a narrative approach and mapped to the three self-care domains (maintenance, monitoring, and management). We summarized patterns across interventions, identified gaps, and highlighted underrepresented factors (e.g., cultural context, psychological well-being). Although most of the included studies were randomized controlled trials, the decision not to weigh the strength of evidence aligns with the scoping review methodology, which prioritizes mapping the breadth of research rather than evaluating its quality.

- Explicitly mentions PRISMA-ScR compliance.
- Adds detail on data charting (development, piloting, refinement, and dual extraction).
- Revises language inclusion criteria per reviewer request.
- Clarifies synthesis approach and rationale for no critical appraisal.

## 3. Results

### 3.1. Literature Selection

**Figure 2:**
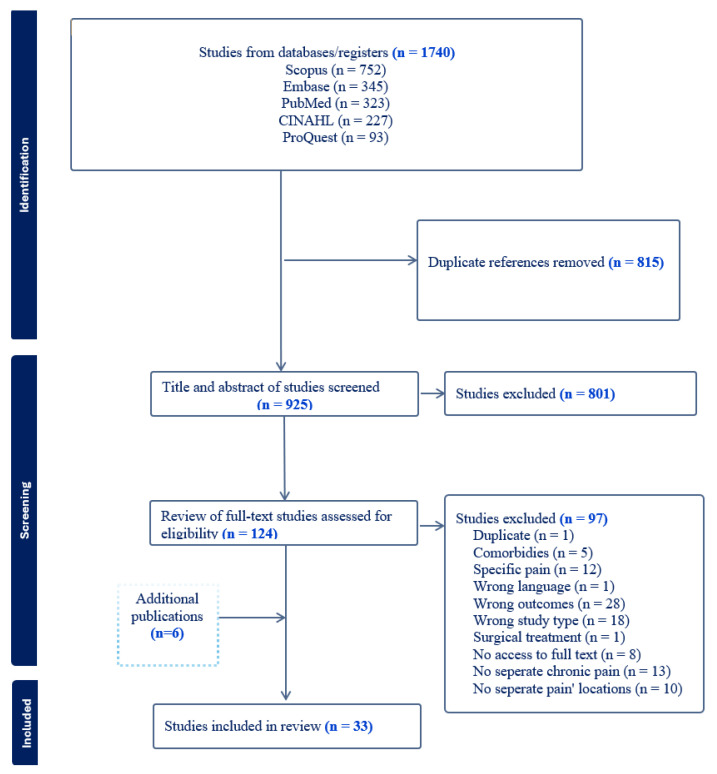
PRISMA flowchart of the literature search and study selection.

The literature search yielded 1740 records. Following the removal of duplicates and a two-stage screening process, 27 studies met the eligibility criteria. An additional six studies were identified through a hand-search of reference lists, resulting in a final sample of 33 studies for synthesis. The full study selection process is detailed in the PRISMA flowchart (Figure 2).

### 3.2. Characteristics of Included Studies

The details of the 33 studies included in the final analysis are presented in Table 1 and Appendix B. These studies were published between 2005 and 2024, with the majority (n = 21) published in the last 10 years. The evidence base is primarily composed of quantitative efficacy trials. The majority of studies were randomized controlled trials (n = 27) focused on chronic neck pain (n = 29), with only two studies employing qualitative methods to explore patient experiences. Other designs were used infrequently, including single-arm trials (n = 2), a two-armed pre-test/post-test design (n = 1), and a three-armed trial (n = 1). Sample size varied widely from 10 to 517, with a median of 64 participants.

This research was geographically concentrated in high-income countries (n = 25), as classified by the World Bank [37]. These countries included the United States, the United Kingdom, Germany, Sweden, Latvia, Spain, Japan, and Hong Kong. Additionally, the study featured a notable gender imbalance, with 27 studies reporting a higher prevalence in females. The typical participant was over 40 years old, reflecting a focus on mid-life to older adult populations. Interventions were most often delivered in healthcare settings (n = 17), though universities, communities, and workplaces also featured. Therapies were split between single-approach (n = 19) and multi-approach (n = 14) formats. The single-approach format involved the use of one therapeutic technique, such as exercise, qigong, massage, thermotherapy, yoga, meditation, or acupuncture. In contrast, multi-approach formats incorporated a combination of different therapeutic techniques. Examples include individualized self-management combined with physical therapy intervention, therapeutic exercises combined with pain neuroscience education, guideline-based physical therapy plus dry needling, osteopathic manipulative treatment associated with exercise, Alexander lessons plus usual care, and acupuncture plus usual care. Pain was the most frequently assessed outcome (n = 26), followed by disability (n = 23), psychological factors (n = 13), such as depression, anxiety, fatigue, or anger, and quality of life (n = 9). Disability was defined through validated self-report measures, including the Neck Disability Index (NDI), the Northwick Park Neck Pain Questionnaire (NPQ), the Copenhagen Neck Functional Disability Scale, the Neck Pain and Disability Questionnaire, or the Neck Pain and Disability Scale (NPAD). Notably, nearly all studies (n = 32) reported positive effects, suggesting a generally favorable impact of interventions across diverse designs and settings.

It is also noted that the predominance of studies from high-income countries and the overrepresentation of women may skew the evidence toward self-care strategies common in these contexts, such as structured exercise or mind–body practices, while approaches relevant to low-resource settings or male populations may remain overlooked. This highlights socioeconomic and gender-related influences on self-care, which are further discussed in Section 4.

**Table 1:** Descriptive data from included studies.

Study Reference Number	Country (Setting)	Study Design (Intervention Group)	Participant Characteristics	Outcomes
**Quantitative/Key Findings: Positive**				
[38]	Hong Kong (physiotherapy outpatient departments)	RCT (exercise)	CNP (N = 145) - Mean age: 43.3/9.7 years - Female: 71.6%	Pain, disability, medication, sick leave, and patient satisfaction
[39]	Sweden (physiotherapy departments in primary care)	RCT (Qigong)	CNP (N = 122) - Mean age: 44.9/12.3 years - Female: 73%	Pain, disability, grip strength, and cervical range of motion
[40]	India (software companies)	RCT (self-SNAGS along with conventional management)	CNP (N = 38) - Computer users - Mean age: 40.8 years - Females: 53%	Incidence of neck pain, range of motion, and disability
[41]	Hong Kong (outpatient physiotherapy department of Kwong Wah Hospital)	RCT (active electrical stimulation of acupuncture points on the wrist + standardized neck exercise)	CNP (N = 49) - No statistically significant differences in baseline characteristics between active and sham groups	Pain
[42]	US (Group Health, nonprofit, integrated healthcare system)	Randomized, parallel-group trial (Therapeutic neck massage)	CNP (N = 64) - Mean age: 47.4 years - Female: 68.8%	Neck-related disability and symptom bothersomeness
[43]	Germany (gymnasia)	RCT (Qigong or exercise therapy)	CNP (N = 123) Qigong: mean age (44.7 ± 10.8), female (85.7%) Exercise Therapy: mean age (44.4 ± 10.9), female (89.7%)	Pain, disability, and quality of life
[44]	Germany (community)	RCT (thermotherapy self-treatment)	CNP (N = 50) - Mean age: 57.18/12.3 years - Female: 74%	Pain, functional disability and health-related quality of life
[45]	India (Department of Musculoskeletal Physiotherapy, Rural Hospital)	RCT (self-sustained natural apophyseal glides (SNAGS), therapist-administered SNAGS)	CNP (N = 112) Self-SNAGs: mean age (33.6 ± 7.36), male/female (15/18) SNAGs: mean age (37.23 ± 9.1), male/female (11/19)	Pain, disability, active cervical range of motion
[46]	Germany (outpatient Department/Hospital Berlin, Department of Internal and Integrative Medicine)	RCT (a 9-week Iyengar yoga program with weekly 90 min classes)	CNP (N = 77) - Mean age: 47.9 ± 7.9 years - Female: 67%	Pain, disability, quality of life, and psychological outcomes
[47]	Germany (single center, Department of Complementary and Integrative Medicine)	RCT (a 9-week yoga course)	CNP (N = 51) - Mean age: 47.8 years - Female: 82.4%	Pain, disability, quality of life, cervical range of motion, proprioceptive acuity, and pressure pain threshold
[48]	Spain (a university research laboratory)	RCT (Group 1: manual therapy and therapeutic patient education/Group 2: Group 1 (content) + a therapeutic exercise protocol)	CNP (N = 45) Group 1: mean age 40.9 (16.2), female (86.7%) Group 2: mean age 39.8 (13.4), female (66.7%)	Disability, fear-avoidance beliefs, neck flexor muscle endurance test, fatigue scale
[49]	Germany (outpatient department, Department of Internal and Complementary Medicine)	RCT (an 8-week meditation program with weekly 90 min classes)	CNP (N = 89) - Mean age: 49.7/10.5 years - Female: 73%	Pain, disability, perceived stress, quality of life and psychological outcomes
[50]	UK (primary care)	RCT (12 acupuncture sessions or 20 one-to-one Alexander lessons + usual care)	CNP (N = 517) - Mean age: 53.2/13.8 years - Female: 69%	Pain, disability, quality of life, and adverse events
[51]	Latvia (public health care service)	RCT (team learning (TL) with self-management (SM) strategies)	CNSP (N = 31); bank and agricultural advisory services VDT employees; - Age: 22–50 - 16 females, 15 males	Pain, quality of life,
[52]	Germany (Department of Complementary and Integrative Medicine)	RCT (Tai Chi or conventional neck exercises)	CNP (N = 114) Taichi: mean age: 52 ± 10.9; female/male: 28/10 Neck exercises: mean age 47 ± 12.3; female/male: 31/6	Pain, disability, quality of life, well-being, and perceived stress, postural and interoceptive awareness, satisfaction, safety
[53]	UK (4 outpatient physiotherapy departments)	RCT (interactive behavioral modification therapy (IBMT))	CNP (N = 57) - No significant differences in baseline demographic scores between the groups	Disability, pain
[54]	US (University of Idaho)	A single-group, multiple-baseline design (ten one-hour group classes in the Alexander technique)	CNP (N = 10) - Mean age: 48/10 years - eight women, two men	Self-reports, superficial neck flexor activation and fatigue, posture
[55]	UK (primary care)	ATLAS randomized, controlled trial (Alexander lessons + usual care, acupuncture + usual care)	CNP (N = 517) - Mean age: 54/14 years - Female: 69%	Pain and disability, self-efficacy, self-care
[56]	China (Department of Rehabilitation Medicine)	Press needle combined with transcutaneous electrical nerve stimulation	CNP (N = 80) - Mean age: 33.3/8.8 years - 28 women, 12 men	Pain and disability, range of motion, neck muscle average electromyography (EMG)
[57]	Brazil (no mention)	A pragmatic RCT (osteopathic manipulative treatment associated with exercise group)	CNP (N = 90) - Mean age: 40.2/12.3 years - Female: 93.3%	Pain, disability, range of motion, fear-avoidance beliefs, and pain self-efficacy
[58]	Spain (no mention)	RCT (a self-MRT (myofascial release therapy) + INYBI device)	CNP (N = 58) - Mean age: 34.6/4.7 years - Females: 77.6%	Pain and active cervical range-of-movement
[59]	Brazil (the community)	RCT (guideline-based physical therapy plus dry needling)	CNP (N = 116) - Mean age: 39.3/9.9 years - Female: 44%	Pain, disability, global perceived effect, quality of sleep, pain catastrophizing, and self-efficacy
[60]	US (University of Idaho)	A two-group, quasi-randomized, pre-test/post-test design (A general Alexander technique class)	CNP (N = 16), mean 6 h/day sitting - Mean age: 51/17 years - 9 women, 7 men	Pain/disability, pain self-efficacy, activation of the sternocleidomastoid muscles, and posture
[61]	Iran (two rehabilitation and physiotherapy centers)	A three-arm RCT (Tterapeutic exercises alone (TE) or combined (therapeutic exercises + PNE-pain neuroscience education))	CNP (N = 72) TE: mean age (31.18 ± 6.37), female (41.66%), male (58.33%) Combined: mean age (33.45 ± 7.08), female (54.16%), male (45.83%)	Pain/disability
[62]	Spain (School of Health Sciences of the University of Granada)	Prospective, parallel group, randomized clinical trial (an individualized self-management combined with physical therapy intervention)	CNP (N = 53) Experimental group: mean age (38.88 ± 14.01) Control group: mean age (40.06 ± 8.32)	Disability, Fear-Avoidance Beliefs, Health-Related Quality of Life, Pain, Anxiety, and Depression
[63]	Iran (universities, university hospitals, and primary care)	Multicenter assessor-blinded RCT (pain neuroscience education (PNE)/face-to-face and online)	CNP (N = 80) - Mean age: 44.5/6.4 years - Female: 55%	Pain and fear of movement
[64]	Japan (Hamamatsu University Hospital Translational Research Unit)	A single-center, single-arm, open-label feasibility study (light-emitting diodes)	CNSP (N =10) - Mean age: 43.7/10.3 years - 2 males and 8 females	Neck and shoulder stiffness, pain, skin temperature, heart rate variability, and baroreceptor reflex sensitivity
[65]	Brazil (no mention)	RCT (OMT plus EG)	CNP (N = 90) - Mean age: 40.2/12.3 years - Female: 93.3%	Pain, disability, range of motion, fear-avoidance beliefs, and pain self-efficacy
[66]	Japan (no mention)	Open-label trial (2-week self-care with gentle mechanical skin stimulation)	Chronic neck and shoulder discomfort (N = 12) - Mean age: 47 years - 7 females and 5 males	Pain sensation, discomfort, and difficulty in moving; joint range of motions
[67]	Japan (a medical school and a medical institution)	RCT (acupuncture using press needles)	CNP (N = 50) - Mean age: 33.1/11.1 years - Female: 62%	Pain, disability, pressure pain threshold
**Quantitative/Key findings: Neutral effect**				
[68]	Germany (universities, gyms, general practitioners ’offices, and local subways)	Pragmatic randomized trial (app-based relaxation exercises)	CNP (N = 220) - Mean age: 38.9/11.3 years - Female: 67.3%	Pain, pain-related stress, sick-leave days, pain medication intake, and adherence
**Qualitative/Key findings: Positive**				
[69]	Sweden (primary healthcare center)	A qualitative evaluation (Feldenkrais groups)	CNSP (N = 14) - Mean age: 44 years - Female: 100%	Experiences, effects of movement exercises, self-practice - Comparison with other methods and opinions
[70]	UK (primary care)	Longitudinal sub-study within the ATLAS trial (Alexander technique lessons OR acu-puncture sessions)	CNP (N = 30)	Perspectives of participants, self-efficacy, self-care

RCT: randomized controlled trial; CNP: chronic neck pain; CNSP: chronic neck and shoulder pain; VDT: video display terminal; IBMT: interactive behavioral modification therapy; OMT: osteopathic manipulative treatment; EG: e group; EMG: electromyography; SNAGs: sustained natural apophyseal glides.

### 3.3. Self-Care Concepts and Self-Care Behaviors

The interventions from the 33 included studies were analyzed using the self-care framework of maintenance, monitoring, and management [30,31,32]. As shown in Table 2, Appendix B and Appendix C, this synthesis revealed a clear pattern in how TCAM-based self-care is currently addressed in the literature.

**Figure 3:**
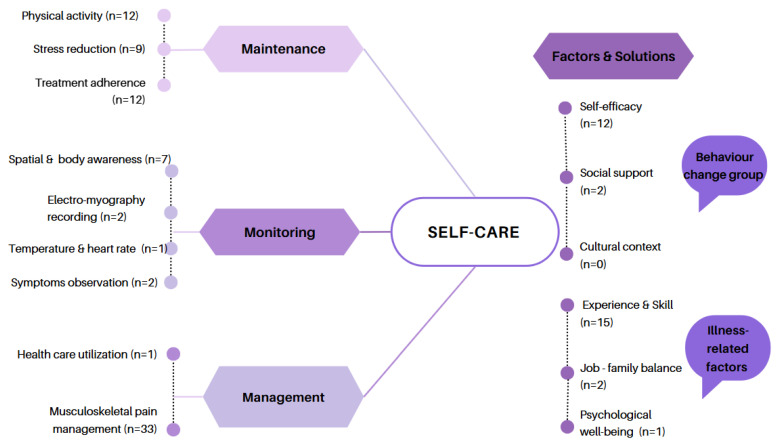
Distribution of self-care concepts and self-care behaviors.

Our analysis showed a strong emphasis on self-care management (present in all 33 studies) and self-care maintenance (n = 20), typically involving pain control and physical activity (Figure 3). In stark contrast, self-care monitoring (n = 11) was significantly underrepresented. These monitoring elements were implemented through techniques including symptom diaries, self-observation, and physiological measurements such as temperature or EMG. A holistic approach integrating all three components was rare, found in only five studies, primarily those investigating mind–body therapies like Tai Chi. Regarding the influencing factors on self-care, “Experience and Skill” (n = 15) and “Self-efficacy” (n = 12) were the most frequently examined. However, other critical factors identified by Riegel’s theory, such as social support (n = 2), job–family balance (n = 2), cultural context (n = 0), and psychological well-being (n = 1), were almost entirely overlooked.

## 4. Discussion

This scoping review aimed to explore how traditional, complementary, and alternative medicine (TCAM) contributes to self-care among patients with chronic neck and shoulder pain. Utilizing the middle-range theory of self-care of chronic illness as an analysis framework, we conducted a gap analysis across its three domains: self-care maintenance, monitoring, and management. Our review found that the ‘self-care monitoring’ component is largely absent, despite being a vital element in the daily self-care of chronic patients [71]. This essential domain could provide significant information for decision-making in future actions [72]. While all studies addressed self-care management, consistent with previous reviews [73], our analysis highlights the limited integration of all components. Thus, we recommend designing interventions that holistically incorporate maintenance, monitoring, and management to enhance the heterogeneity of designs and interventions.

Another finding is that not all behaviors suggested by the middle-range theory of self-care of chronic illness have been thoroughly examined. There has been a focus on certain behaviors, such as physical activity and treatment adherence, which are frequently studied, whereas other vital behaviors, such as sleep and nutrition practices, are often overlooked. These behaviors play a significant role in pain management and overall well-being. For example, an individual may use vitamins, medicinal herbs [74], or meditation [75] or seek acupuncture at traditional clinics [76]. On the other hand, risky behaviors such as substance abuse have been linked to increasing pain-related mortality [77], and factors like sleep patterns and smoking status may confound the relationship between TCAM and pain management [78,79].

Additionally, self-care factors such as self-efficacy, experience, and skill have been well-explored in efforts to enhance self-care by fostering individual habits. However, other factors such as cultural beliefs and values have been less examined, despite their influence on treatment preferences. For example, TCAM is more commonly used in Asian culture [80], and minority populations often favor non-invasive medical treatment and self-management for their pain [81]. Our findings also revealed that existing research has predominantly been conducted in high-income countries, leaving regions most affected by chronic neck and shoulder pain, such as Southeast Asia and Africa, significantly underrepresented [39,49,50,67,82]. In fact, culture-based interventions have demonstrated positive outcomes, including lower rates of rehospitalization and mortality, increased patient satisfaction, and cost savings [83]. This highlights the need for tailored interventions that align with patients’ cultural backgrounds and customs. Therefore, another key recommendation from this analysis is to expand research into culturally informed self-care strategies that reflect patients’ values and preferences.

Psychological factors are another area of concern. Despite the high prevalence of stress, anxiety, and depression among individuals with chronic pain [84], few interventions address these dimensions. Given that everyday stress can significantly increase mortality risk in chronically ill adults [85], future self-care programs must incorporate psychological support to improve holistic outcomes. Integrating TCAM-based self-care principles into conventional educational strategies enables healthcare professionals, including nurses, physiotherapists, and primary care physicians, to deliver a more comprehensive, patient-centered approach that promotes engagement, improves adherence, and enhances overall quality of life.

### Limitations

While this review employed a rigorous methodology, certain limitations should be acknowledged. Initially, the inclusion criteria restricted studies to those published in English, which may have led to the omission of relevant evidence from non-English sources. Following reviewer feedback, we have expanded eligibility to include studies published in other languages if they provide an English abstract and sufficient methodological detail for data extraction. Nevertheless, this approach may still underrepresent culturally diverse perspectives and interventions related to cultural groups and contextual backgrounds, particularly from regions where TCAM practices are prevalent, such as Southeast Asia and Africa. Future reviews should incorporate multilingual searches and translation strategies to ensure a more comprehensive synthesis of global evidence.

It should be noted that the included studies did not adopt a globally standardized definition of disability. Instead, most relied on self-report scales validated for musculoskeletal conditions. While these tools are widely accepted in clinical research, variability across countries may introduce conceptual differences. This heterogeneity represents a limitation and underscores the need for future research to consider harmonized definitions of disability to improve comparability.

## 5. Conclusions

This scoping review systematically mapped the evidence on TCAM-based self-care strategies for adults with chronic neck and shoulder pain. Current research predominantly focuses on self-care maintenance (e.g., physical activity and treatment adherence) and management (e.g., pain relief), while self-care monitoring and broader psychosocial and cultural dimensions remain underexplored. These gaps highlight the need for future research to prioritize the design of TCAM self-care interventions that explicitly integrate monitoring and psychosocial support.

Given the exploratory nature of scoping reviews, our findings should not be interpreted as prescriptive recommendations for clinical practice or policy. Instead, they provide a foundation for identifying research priorities, such as the following:

- Developing interventions that incorporate all three self-care components.
- Examining cultural influences and psychological well-being in TCAM-based self-care.
- Expanding research beyond high-income countries to ensure global relevance.

By addressing these gaps, future studies can contribute to more comprehensive and patient-centered strategies for managing chronic neck and shoulder pain.

## Figures and Tables

**Table 2:** Analysis of self-care concepts and behaviors identified in single- and multi-approach therapies.

Study (Year)	Self-Care Concepts			Self-Care Behaviors
	Maintenance	Monitoring	Management	
**Single-Approach Therapies**				
[38]	- Physical activity	X	- Musculoskeletal pain management	- Experience and skill
[39]	- Stress reduction - Physical activity - Treatment adherence	X	- Musculoskeletal pain management	X
[42]	X	X	- Musculoskeletal pain management	- Experience and skill
[44]	- Treatment adherence	X	- Musculoskeletal pain management	X
[45]	- Treatment adherence	X	- Musculoskeletal pain management	X
[46]	X	X	- Musculoskeletal pain management	X
[47]	- Physical activity - Treatment adherence	X	- Musculoskeletal pain management	X
[49]	- Stress reduction	X	- Musculoskeletal pain management	- Experience and skill
[52]	- Stress reduction - Physical activity - Treatment adherence	- Spatial and Body awareness	- Musculoskeletal pain management	- Experience and skill
[53]	- Stress reduction - Physical activity	X	- Musculoskeletal pain management	- Self-efficacy
[54]	X	- Spatial and body awareness - Electromyography recording	- Musculoskeletal pain management	- Self-efficacy
[58]	X	X	- Musculoskeletal pain management	X
[60]	X	- Spatial and body awareness	- Musculoskeletal pain management	- Self-efficacy - Experience and skill
[63]	X	X	- Musculoskeletal pain management	- Experience and skill
[64]	X	- Temperature and heart rate	- Musculoskeletal pain management	X
[68]	- Stress reduction - Physical activity - Treatment adherence	X	- Musculoskeletal pain management	- Experience and skill
[67]	X	X	- Musculoskeletal pain management	- Experience and skill
[69]	X	- Spatial and body awareness	- Musculoskeletal pain management	- Experience and Skill
[70]	X	- Spatial and body awareness	- Musculoskeletal pain management	- Self-efficacy - Experience and skill - Support from others
**Multi-approach therapies**				
[40]	- Physical activity	X	- Musculoskeletal pain management	X
[41]	X	X	- Musculoskeletal pain management	- Self-efficacy
[43]	- Physical activity	X	- Musculoskeletal pain management	- Experience and skill
[48]	- Stress reduction - Physical activity - Treatment adherence	X	- Musculoskeletal pain management	- Self-efficacy
[50]	- Treatment adherence	- Spatial and body awareness	- Musculoskeletal pain management	- Self-efficacy - Experience and skill
[51]	- Stress reduction	X	- Musculoskeletal pain management	- Job and family life - Support from Others
[55]	- Treatment adherence	- Spatial and body awareness	- Healthcare utilization - Musculoskeletal pain management	- Self-efficacy
[56]	X	- Electromyography recording	- Musculoskeletal pain management	X
[57]	- Stress reduction - Physical activity	X	- Musculoskeletal pain management	- Self-efficacy - Mood states
[59]	- Physical activity	X	- Musculoskeletal pain management	- Self-efficacy - Experience and skill
[61]	X	X	- Musculoskeletal pain management	- Self-efficacy - Experience and skill
[62]	- Stress reduction - Treatment adherence	X	- Musculoskeletal pain management	- Experience and skill
[65]	- Physical activity - Treatment adherence	- Symptoms observation	- Musculoskeletal pain management	- Self-efficacy - Job and family life
[66]	- Treatment adherence	- Symptoms observation	- Musculoskeletal pain management	X

## Data Availability

No new data were created or analyzed in this study. Data sharing is not applicable to this article.

## Supplementary Materials

The following supporting information can be downloaded at https://www.mdpi.com/article/10.3390/nursrep16020044/s1, Supplementary File S1: Prisma checklist.

## Public Involvement Statement

No public involvement in any aspect of this research.

## Guidelines and Standards Statement

This manuscript was drafted against the Preferred Reporting Items for Systematic Reviews and Meta-Analyses extension for Scoping Reviews (PRISMA-ScR) [27].

## Abbreviations

The following abbreviations are used in this manuscript:

AT	Alexander Technique
CINAHL	Cumulative Index to Nursing and Allied Health Literature
CNP	Chronic Neck Pain
CNSP	Chronic Neck and Shoulder Pain
EG	Exercise Group
IBMT	Interactive Behavioral Modification Therapy
JBI	Joanna Briggs Institute
LEDs	Light-Emitting Diodes
MRT	Myofascial Release Therapy
OMT	Osteopathic Manipulative Treatment
PBE	Pain Biomechanics Education
PICO	Population, Intervention, Comparison, and Outcomes
PNE	Pain Neuroscience Education
PNEP	Progressive Neck Exercise Program
PRISMA-ScR	Preferred Reporting Items for Systematic Reviews and Meta-Analyses-Extension for Scoping Reviews
QoL	Quality of Life
RCT	Randomized Controlled Trial
TCAM	Traditional, Complementary, and Alternative Medicine
TE	Therapeutic Exercises

## Appendix A. Search Strategy

**Database**	**Search Term**
**Pubmed**	
#1	“neck pain*” [Title/Abstract] OR “shoulder pain” [Title/Abstract] OR neckache*[Title/Abstract] OR “neck ache*” [Title/Abstract] OR “neck tension” [Title/Abstract] OR “cervical pain*” [Title/Abstract] OR “subacromial pain” [Title/Abstract] OR cervicalgia* [Title/Abstract] OR cervicodynia*[Title/Abstract] OR “neck disorder*” [Title/Abstract] OR “shoulder disorder*” [Title/Abstract] OR “ musculoskeletal pain*” [Title/Abstract] OR “musculoskeletal disorder*” [Title/Abstract] OR “neck shoulder” [Title/Abstract:~2]
#2	“Neck Pain” [Mesh]
#3	“Shoulder Pain” [Mesh]
#4	#1 OR #2 OR #3
#5	“complementary medicine*” [Title/Abstract] OR “complementary therap*” [Title/Abstract] OR “complementary healthcare” [Title/Abstract] OR “alternative healthcare” [Title/Abstract] OR “traditional medicine*” [Title/Abstract] OR “traditional therap*” [Title/Abstract] OR “traditional healthcare” [Title/Abstract] OR “alternative medicine*” [Title/Abstract] OR “alternative therap*” [Title/Abstract] OR “integrative medicine*” [Title/Abstract] OR “integrative therap*” [Title/Abstract] OR “integrative healthcare” [Title/Abstract] OR “complementary alternative” [Title/Abstract:~2] OR “complementary integrative “[Title/Abstract:~2] OR “Chinese medicine*” [Title/Abstract] OR “Ayurvedic medicine*” [Title/Abstract] OR acupuncture[Title/Abstract] OR electroacupuncture [Title/Abstract] OR auriculotherap*[Title/Abstract] OR meridian*[Title/Abstract] OR moxibustion [Title/Abstract] OR “cupping therap*” [Title/Abstract] OR “dry needling” [Title/Abstract] OR “holistic health” [Title/Abstract] OR “bioresonance therap*” [Title/Abstract] OR homeopathy [Title/Abstract] OR “horticultural therap*” [Title/Abstract] OR “mind-body therap*” [Title/Abstract] OR aromatherap*[Title/Abstract] OR biofeedback [Title/Abstract] OR “breathing exercise*” [Title/Abstract] OR hypnosis [Title/Abstract] OR imagery [Title/Abstract] OR psychotherap*[Title/Abstract] OR “laughter therap*” [Title/Abstract] OR meditation [Title/Abstract] OR “mental healing” [Title/Abstract] OR psychodrama [Title/Abstract] OR “relaxation therap*” [Title/Abstract] OR “massage therap*” [Title/Abstract] OR “tai ji” [Title/Abstract] OR “therapeutic touch” [Title/Abstract] OR yoga [Title/Abstract] OR “musculoskeletal manipulation*” [Title/Abstract] OR kinesiology [Title/Abstract] OR chiropractic [Title/Abstract] OR osteopathic [Title/Abstract] OR “soft tissue therap*” [Title/Abstract] OR organotherap*[Title/Abstract] OR “art therap*” [Title/Abstract] OR “color therap*” [Title/Abstract] OR “dance therap*” [Title/Abstract] OR “music therap*” [Title/Abstract] OR “play therap*” [Title/Abstract] OR naturopath*[Title/Abstract] OR phytotherap*[Title/Abstract] OR prolotherap*[Title/Abstract] OR reflexotherap*[Title/Abstract] OR “spiritual therap*” [Title/Abstract] OR therap*[Title/Abstract]
#6	“Complementary Therapies” [Mesh] OR “Integrative Medicine” [Mesh] OR “Medicine, Traditional” [Mesh]
#7	#5 OR #6
#8	self-care[Title/Abstract] OR “self care” [Title/Abstract] OR self-manage*[Title/Abstract] OR “self manage*” [Title/Abstract] OR “self administration” [Title/Abstract] OR “patient managed” [Title/Abstract] OR self-treatment[Title/Abstract] OR “self treatment” [Title/Abstract] OR “self efficacy” [Title/Abstract] OR self-efficacy[Title/Abstract]
#9	“Self Care” [Mesh]
#10	#8 OR #9
#11	#4 AND #7 AND #10
**Scopus**	**Search within article title, abstract, keywords**
#1	“neck pain*” OR “shoulder pain” OR neckache* OR “neck ache*” OR “neck tension” OR “cervical pain*” OR “subacromial pain” OR cervicalgia* OR cervicodynia* OR “neck disorder*” OR “shoulder disorder*” OR “musculoskeletal pain*” OR “musculoskeletal disorder*” OR neck W/2 shoulder
#2	“complementary medicine*” OR “complementary therap*” OR “complementary healthcare” OR “alternative healthcare” OR “traditional medicine*” OR “traditional therap*” OR “traditional healthcare” OR “alternative medicine*” OR “alternative therap*” OR “integrative medicine*” OR “ integrative therap*” OR “integrative healthcare” OR complementary W/2 alternative OR complementary W/2 integrative OR acupuncture OR electroacupuncture OR auriculotherap* OR meridian* OR moxibustion OR “cupping therap*” OR “dry needling” OR “holistic health” OR “bioresonance therap*” OR homeopathy OR “horticultural therap*” OR “mind-body therap*” OR aromatherap* OR biofeedback OR “breathing exercise*” OR hypnosis OR imagery OR psychotherap* OR “laughter therap*” OR meditation OR “mental healing” OR psychodrama OR “relaxation therap*” OR “massage therap*” OR “tai ji” OR “therapeutic touch” OR yoga OR “musculoskeletal manipulation*” OR kinesiology OR chiropractic OR osteopathic OR “soft tissue therap*” OR organotherap* OR “art therap*” OR “color therap*” OR “dance therap*” OR “music therap*” OR “play therap*” OR naturopath* OR phytotherap* OR prolotherap* OR reflexotherap* OR “spiritual therap*” OR therap*
#3	self-care OR “self care” OR self-manage* OR “self manage*” OR “self administration” OR “patient managed” OR self-treatment OR “self treatment” OR self-efficacy OR “self efficacy”
**Embase**	
#1	‘neck pain*’:ti,ab OR ‘shoulder pain’:ti,ab OR neckache*:ti,ab OR ‘neck ache*’:ti,ab OR ‘neck tension’:ti,ab OR ‘cervical pain*’:ti,ab OR ‘subacromial pain’:ti,ab OR cervicalgia*:ti,ab OR cervicodynia*:ti,ab OR ‘neck disorder*’:ti,ab OR ‘shoulder disorder*’:ti,ab OR ‘musculoskeletal pain*’:ti,ab OR ‘musculoskeletal disorder*’:ti,ab OR neck NEAR/2 shoulder:ti,ab
#2	‘neck pain’/exp OR ‘shoulder pain’/exp
#3	#1 OR #2
#4	(‘complementary medicine*’:ti,ab OR ‘complementary therap*’:ti,ab OR ‘complementary healthcare’:ti,ab OR ‘alternative healthcare’:ti,ab OR ‘traditional medicine*’:ti,ab OR ‘traditional therap*’:ti,ab OR ‘traditional healthcare’:ti,ab OR ‘alternative medicine*’:ti,ab OR ‘alternative therap*’:ti,ab OR ‘integrative medicine*’:ti,ab OR ‘integrative therap*’:ti,ab OR ‘integrative healthcare’:ti,ab OR complementary NEAR/2 alternative:ti,ab OR complementary NEAR/2 integrative:ti,ab) OR acupuncture:ti,ab OR electroacupuncture:ti,ab OR auriculotherap*:ti,ab OR meridian*:ti,ab OR moxibustion:ti,ab OR ‘cupping therap*’:ti,ab OR ‘dry needling’:ti,ab OR ‘holistic health’:ti,ab OR ‘bioresonance therap*’:ti,ab OR homeopathy:ti,ab OR ‘horticultural therap*’:ti,ab OR ‘mind-body therap*’:ti,ab OR aromatherap*:ti,ab OR biofeedback:ti,ab OR ‘breathing exercise*’:ti,ab OR hypnosis:ti,ab OR imagery:ti,ab OR psychotherap*:ti,ab OR ‘laughter therap*’:ti,ab OR meditation:ti,ab OR ‘mental healing’:ti,ab OR psychodrama:ti,ab OR ‘relaxation therap*’:ti,ab OR ‘massage therap*’:ti,ab OR ‘tai ji’:ti,ab OR ‘therapeutic touch’:ti,ab OR yoga:ti,ab OR ‘musculoskeletal manipulation*’:ti,ab OR kinesiology:ti,ab OR chiropractic:ti,ab OR osteopathic:ti,ab OR ‘soft tissue therap*’:ti,ab OR organotherap*:ti,ab OR ‘art therap*’:ti,ab OR ‘color therap*’:ti,ab OR ‘dance therap*’:ti,ab OR ‘music therap*’:ti,ab OR ‘play therap*’:ti,ab OR naturopath*:ti,ab OR phytotherap*:ti,ab OR prolotherap*:ti,ab OR reflexotherap*:ti,ab OR ‘spiritual therap*’:ti,ab OR therap*:ti,ab
#5	‘alternative medicine’/exp OR ‘integrative medicine’/exp OR ‘traditional medicine’/exp
#6	#4 OR #5
#7	self-care:ti,ab OR ‘self care’:ti,ab OR self-manage*:ti,ab OR ‘self manage*’:ti,ab OR ‘self administration’:ti,ab OR ‘patient managed’:ti,ab OR self-treatment:ti,ab OR ‘self treatment’:ti,ab OR self-efficacy:ti,ab OR “self efficacy”:ti,ab
#8	‘self care’/exp
#9	#7 OR #8
#10	#3 AND #6 AND #9
**Cinahl Complete (EBSCOhost)**	
S1	TI (“neck pain*” OR “shoulder pain” OR “neck and shoulder pain” OR neckache* OR “neck ache*” OR “neck tension” OR “cervical pain*” OR “subacromial pain” OR cervicalgia* OR cervicodynia* OR “neck disorder*” OR “shoulder disorder*” OR “ musculoskeletal pain*” OR “musculoskeletal disorder*”) OR AB (“neck pain*” OR “shoulder pain” OR “neck and shoulder pain” OR neckache* OR “neck ache*” OR “neck tension” OR “cervical pain*” OR “subacromial pain” OR cervicalgia* OR cervicodynia* OR “neck disorder*” OR “shoulder disorder*” OR “ musculoskeletal pain*” OR “musculoskeletal disorder*”) OR neck N2 shoulder
S2	(MH “Neck Pain”) OR (MH “Shoulder Pain”)
S3	S1 OR S2
S4	TI (“complementary medicine*” OR “complementary therap*” OR “complementary healthcare” OR “alternative healthcare” OR “traditional medicine*” OR “traditional therap*” OR “traditional healthcare” OR “alternative medicine*” OR “alternative therap*” OR “integrative medicine*” OR “ integrative therap*” OR “integrative healthcare” OR complementary N2 alternative OR complementary N2 integrative OR acupuncture OR electroacupuncture OR auriculotherap* OR meridian* OR moxibustion OR “cupping therap*” OR “dry needling” OR “holistic health” OR “bioresonance therap*” OR homeopathy OR “horticultural therap*” OR “mind-body therap*” OR aromatherap* OR biofeedback OR “breathing exercise*” OR hypnosis OR imagery OR psychotherap* OR “laughter therap*” OR meditation OR “mental healing” OR psychodrama OR “relaxation therap*” OR “massage therap*” OR “tai ji” OR “therapeutic touch” OR yoga OR “musculoskeletal manipulation*” OR kinesiology OR chiropractic OR osteopathic OR “soft tissue therap*” OR organotherap* OR “art therap*” OR “color therap*” OR “dance therap*” OR “music therap*” OR “play therap*” OR naturopath* OR phytotherap* OR prolotherap* OR reflexotherap* OR “spiritual therap*” OR therap*) OR AB (“complementary medicine*” OR “complementary therap*” OR “complementary healthcare” OR “alternative healthcare” OR “traditional medicine*” OR “traditional therap*” OR “traditional healthcare” OR “alternative medicine*” OR “alternative therap*” OR “integrative medicine*” OR “ integrative therap*” OR “integrative healthcare” OR complementary N2 alternative OR complementary N2 integrative OR acupuncture OR electroacupuncture OR auriculotherap* OR meridian* OR moxibustion OR “cupping therap*” OR “dry needling” OR “holistic health” OR “bioresonance therap*” OR homeopathy OR “horticultural therap*” OR “mind-body therap*” OR aromatherap* OR biofeedback OR “breathing exercise*” OR hypnosis OR imagery OR psychotherap* OR “laughter therap*” OR meditation OR “mental healing” OR psychodrama OR “relaxation therap*” OR “massage therap*” OR “tai ji” OR “therapeutic touch” OR yoga OR “musculoskeletal manipulation*” OR kinesiology OR chiropractic OR osteopathic OR “soft tissue therap*” OR organotherap* OR “art therap*” OR “color therap*” OR “dance therap*” OR “music therap*” OR “play therap*” OR naturopath* OR phytotherap* OR prolotherap* OR reflexotherap* OR “spiritual therap*” OR therap*)
S5	(MH “Alternative Therapies+”) OR (MH “Alternative Medical Systems+”)
S6	S4 OR S5
S7	TI (self-care OR “self care” OR self-manage* OR “self manage*” OR “self administration” OR “patient managed” OR self-treatment OR “self treatment” OR self-efficacy OR “self efficacy”) OR AB (self-care OR “self care” OR self-manage* OR “self manage*” OR “self administration” OR “patient managed” OR self-treatment OR “self treatment” OR self-efficacy OR “self efficacy”)
S8	(MH “Self Care”) OR (MH “Self Administration+”)
S9	S7 OR S8
S10	S3 AND S6 AND S9
**Public Health Database (ProQuest)**	
S1	“neck pain*” OR “shoulder pain” OR “neck and shoulder pain” OR neckache* OR “neck ache*” OR “neck tension” OR “cervical pain*” OR “subacromial pain” OR cervicalgia* OR cervicodynia* OR “neck disorder*” OR “shoulder disorder*” OR “ musculoskeletal pain*” OR “musculoskeletal disorder*” OR neck NEAR/2 shoulder
S2	mainsubject.Exact(“neck pain” OR “shoulder pain”)
S3	S1 OR S2
S4	“complementary medicine*” OR “complementary therap*” OR “complementary healthcare” OR “alternative healthcare” OR “traditional medicine*” OR “traditional therap*” OR “traditional healthcare” OR “alternative medicine*” OR “alternative therap*” OR “integrative medicine*” OR “ integrative therap*” OR “integrative healthcare” OR complementary NEAR/2 alternative OR complementary NEAR/2 integrative OR acupuncture OR electroacupuncture OR auriculotherap* OR meridian* OR moxibustion OR “cupping therap*” OR “dry needling” OR “holistic health” OR “bioresonance therap*” OR homeopathy OR “horticultural therap*” OR “mind-body therap*” OR aromatherap* OR biofeedback OR “breathing exercise*” OR hypnosis OR imagery OR psychotherap* OR “laughter therap*” OR meditation OR “mental healing” OR psychodrama OR “relaxation therap*” OR “massage therap*” OR “tai ji” OR “therapeutic touch” OR yoga OR “musculoskeletal manipulation*” OR kinesiology OR chiropractic OR osteopathic OR “soft tissue therap*” OR organotherap* OR “art therap*” OR “color therap*” OR “dance therap*” OR “music therap*” OR “play therap*” OR naturopath* OR phytotherap* OR prolotherap* OR reflexotherap* OR “spiritual therap*” OR therap*
S5	mainsubject.Exact(“integrative medicine” OR “traditional medicine” OR “alternative medicine”)
S6	S4 OR S5
S7	self-care OR “self care” OR self-manage* OR “self manage*” OR “self administration” OR “patient managed” OR self-treatment OR “self treatment” OR self-efficacy OR “self efficacy”
S8	mainsubject.Exact(“self care”)
S9	S7 OR S8
S10	S3 AND S6 AND S9
*: a truncation symbol.	

## Appendix B. Study Characteristics

**Reference Number, Country**	**Study Characteristics**			**Setting**	**Intervention Group**	**Control Group**	**Outcomes**	**Key Findings**
	**Study Design**	**Population,** **Sample Size**	**Participant Characteristics**					
**Quantitative**								
[38] Hong Kong	RCT	Chronic neck pain N = 145	Exercise - Mean age: 43.3/9.7 years - Female: 71.6%	Physiotherapy outpatient departments	Exercise	Non-exercise	Pain, disability, medication, sick leave, and patient satisfaction	At week 6, patients with chronic neck pain can benefit from the neck exercise program with significant improvement in disability, pain, and isometric neck muscle strength in different directions. However, the effect of exercise was less favorable at 6 months.
[39] Sweden	RCT	Chronic neck pain N = 122	- Mean age: 44.9/12.3 years - Female: 73%	Physiotherapy departments in primary care	Qigong	Exercise therapy	Pain, disability, grip strength, and cervical range of motion	Both groups reduce pain and disability.
[40] India	RCT	Chronic neck pain N = 38	Computer users, mean age: 40.8 years, 53% females, and 47% males	Software companies	Self-SNAGS along with conventional management	Conventional management: hot packs and advised on correct posture	Incidence of neck pain, range of motion, and disability	Group receiving self-SNAGS showed better carry-over effect during the treatment phase and more during follow-up phase.
[41] Hong Kong	A randomized, sham-controlled trial	Chronic neck pain N = 49	No statistically significant differences in baseline characteristics between the active and sham acustimulation groups	The outpatient physiotherapy department of Kwong Wah Hospital	Active electrical stimulation of acupuncture points on the wrist in addition to the standardized neck exercise	Sham electrical stimulation of acupuncture points on the wrist in addition to the standardized neck exercise	Pain	Significant improvements in physical and psychological well-being when compared with sham.
[42] US	Randomized, parallel-group trial	Chronic neck pain N = 64	- Mean age: 47.4 years - Female: 68.8%	Group Health, a nonprofit, integrated healthcare system	Therapeutic neck massage	A self-care book	neck-related disability and symptom bothersomeness	Massage is safe and may have clinical benefits for treating chronic neck pain, at least in the short term.
[43] Germany	RCT	Chronic neck pain N = 123	- Qigong: mean age (44.7 ± 10.8), female (85.7%) - Exercise Therapy: mean age (44.4 ± 10.9), female (89.7%)	Gymnasia	Qigong or exercise therapy	No treatment	Pain, disability, and quality of life (QoL)	Qigong was more effective than no treatment in patients with chronic neck pain.
[44] Germany	RCT	Chronic neck pain N = 50	- Mean age: 57.18/12.3 years - Female: 74%	Community	Thermotherapy self-treatment	Self-directed usual care	Pain, functional disability and health-related QoL	Effective in relieving pain and improving sensory functioning.
[45] India	RCT	Non-specific CNP N = 112	Self-SNAGs: mean age (33.6 ±7.36), male/female (15/18) SNAGs: mean age (37.23 ± 9.1), male/female (11/19)	Department of Musculoskeletal Physiotherapy, Pravara Rural Hospital (tertiary hospital)	Self-SNAGs, therapist-administered SNAGs	Conventional physiotherapy	Pain, disability, Active Cervical Range of Motion	No significant difference in self administered SNAGS and therapist-administered SNAGS.
[46] Germany	A pilot RCT	Chronic neck pain N = 77	- Mean age: 47.9 ± 7.9 years - Female: 67%	The outpatient department of the Immanuel Hospital Berlin, Department of Internal and Integrative Medicine	A 9-week Iyengar yoga program with weekly 90 min classes	A self-care/ exercise program	Pain, disability, QOL, and psychological outcomes	- Both programs were well tolerated. - Yoga effective treatment with possible additional effects on psychological well-being and QOL.
[47] Germany	RCT	Chronic neck pain N = 51	- Mean age: 47.8 years - Female: 82.4%	Single center, the Department of Complementary and Integrative Medicine	A 9-week yoga course	A self-care manual on home-based exercises	Pain, disability, QoL, cervical range of motion, proprioceptive acuity, and pressure pain threshold	Yoga was more effective: reduced neck pain Intensity, disability, and improved health-related quality of life; improved physiological measures of neck pain.
[48] Spain	RCT	Chronic neck pain N = 45	Group 1: mean age 40.9 (16.2), female (86.7%) Group 2: mean age 39.8 (13.4), female (66.7%)	A university research laboratory	Group 1: manual therapy and therapeutic patient education Group 2: manual therapy, therapeutic patient education, and therapeutic exercise protocol	Manual therapy	Disability, fear-avoidance beliefs, neck flexor muscle endurance test, fatigue scale	Differences between the experimental groups and the control group in the short and medium term. A multimodal treatment is a good method for reducing disability in patients with non-specific chronic neck pain in the short and medium term.
[49] Germany	RCT	Chronic neck pain N = 89	- Mean age: 49.7/10.5 years - Female: 73%	The outpatient department of the Immanuel Krankenhaus Berlin, Department of Internal and Complementary Medicine	An 8-week meditation program (jyoti meditation) with weekly 90 min classes	A home-based exercise program	Pain, disability, perceived stress, QOL, and psychological outcomes	Meditation may support chronic pain patients in pain reduction and pain coping.
[50] UK	RCT	Non-specific CNP N = 517	- Mean age: 53.2/13.8 years - Female: 69%	Primary care	12 acupuncture sessions or 20 one-to-one Alexander lessons (both 600 min total) plus usual care	Usual care alone	Pain, disability, QoL, and adverse events	Acupuncture sessions and Alexander Technique lessons both led to significant reductions in neck pain and associated disability compared with usual care at 12 months. Enhanced self-efficacy may partially explain why longer-term benefits were sustained.
[51] Latvia	RCT	Chronic neck–shoulder pain N = 31	Bank and agricultural advisory services VDT employees; females = 16, males = 15; age between 22 and 50 Vdt: video display terminal	Public healthcare service	Team learning (TL) with self-management (SM) strategies		Pain, QoL	Decrease in neck–shoulder pain intensity, and positive QoL changes.
[52] Germany	A randomized controlled trial	Non-specific CNP N = 114	Taichi: - mean age: 52 ± 10.9 - female/male: 28/10 Neck exercises: - mean age: 47 ± 12.3 - female/male: 31/6	Department of Complementary and Integrative Medicine	Tai Chi or conventional neck exercises	Wait list	Pain and disability, QoL, well-being, and perceived stress, postural and interoceptive awareness, satisfaction, and safety	Tai Chi exercises and conventional neck exercises are equally effective in improving pain and QoL.
[53] UK	Multicenter RCT	Non-specific CNP N = 57	No significant differences in baseline demographic scores between the groups	4 outpatient physiotherapy departments	IBMT	PNEP	Disability, pain	- Both: effective in reducing disability but no significant differences in disability at six-month follow-up. -IBMT: larger increases in functional self-efficacy beliefs, greater reductions in pain intensity and pain-related fear, greater number of participants reporting an MCIC in disability and pain.
[54] US	A single-group, multiple-baseline design	Chronic neck pain N = 10	Eight women, two men; age 48 ± 10 years	University of Idaho	Ten one-hour group classes in AT		self-reports, superficial neck flexor activation and fatigue; posture	A cost-effective approach to reducing neck pain by teaching participants to decrease excessive habitual muscle contraction during everyday activity.
[55] UK	ATLAS RCT	Non-specific CNP N = 517	Mean age: 54/14 years Female: 69%	Primary care	Alexander lessons plus usual care, acupuncture plus usual care	Usual care alone	Pain and disability, self-efficacy, self-care	Alexander Technique lessons led to long-term improvements in the way participants lived their daily lives and managed their neck pain. Alexander lessons promote self-efficacy and self-care, with consequent reductions in chronic neck pain.
[56] China	RCT	Non-specific CNP N = 80	- Mean age: 33.3 ± 8.8 - Female/male: 28/12	Department of Rehabilitation Medicine of the Affiliated Hospital of Southwest Medical University	Press needle combined with transcutaneous electrical nerve stimulation	Transcutaneous electrical nerve stimulation only	Pain and disability, range of motion, neck muscle average electromyography (EMG)	More effective on non-specific neck pain than transcutaneous electrical nerve stimulation only, and no adverse reaction was observed.
[57] Brazil	A pragmatic RCT	Non-specific CNP N = 90	- Mean age: 40.2/12.3 years - Female: 93.3%	No mention	Osteopathic manipulative treatment associated with the exercise group(OMT/EG)	Exercise group (EG)	Pain, disability, range of motion, fear-avoidance beliefs, and pain self-efficacy	OMT/EG reduces pain and improves functional disability more than exercise alone.
[58] Spain	RCT	Non-specific CNP N = 58	- Mean age: 34.6/4.7 years - Females: 77.6%	No mention	Underwent a self-MRT (myofascial release therapy) intervention using the INYBI device	MRT of the suboccipital muscles was performed using the suboccipital muscle inhibition technique	Pain and active cervical range-of-movement	Both are equally effective in enhancing self-reported pain intensity and local pressure pain sensitivity. For cervical mobility, MRT appears to be slightly superior in achieving improvements.
[59] Brazil	RCT	Chronic neck pain N = 116	A group: mean age (39.3 ± 9.9), female (44%) B group: mean age (36.9 ± 11.5), female (40%)	The community	Guideline-based physical therapy plus dry needling (A group)	Guideline-based physical therapy (B group)	Pain, disability, global perceived effect, quality of sleep, pain catastrophizing, and self-efficacy	Small improvements in pain only at 1 month post-randomization, and no effect on disability.
[60] US	A two-group, quasi-randomized, pre-test/post-test design	Chronic neck pain N = 16	9 women, 7 men, mean age (51 ± 17), mean 6 h/day sitting	University of Idaho	A General Alexander technique class	An exercise class	Pain/disability, pain self-efficacy, activation of the sternocleidomastoid muscles, and posture	Both groups reported decreased neck pain/disability after the interventions. Sternocleidomastoid activation decreased only in the Alexander group.
[61] Iran	A three-arm randomized controlled trial	Non-specific CNP N = 72	TE: mean age (31.18 ± 6.37), female (41.66%), male (58.33%) Combined: mean age (33.45 ± 7.08), female (54.16%), male (45.83%)	Two rehabilitation and physiotherapy centers	Therapeutic exercises alone (TE) or combined (therapeutic exercises + PNE-pain neuroscience education)	Did not receive any intervention, but was instructed to maintain the proper position at work and home (brochures); received a comprehensive rehabilitation program after the intervention period	Pain/disability	Pain and disability, fear-avoidance beliefs, and pain catastrophizing were reduced, and pain self-efficacy increased. Both intervention groups displayed more significant effects than the control group, and the combined training group was better than therapeutic exercises alone.
[62] Spain	Prospective, parallel group, randomized clinical trial	Chronic neck pain N = 53	Experimental group: mean age (38.88 ± 14.01) control group: mean age (40.06 ± 8.32)	School of Health Sciences of the University of Granada	An individualized self-management combined with physical therapy intervention	A physical therapy intervention	Disability, fear-avoidance beliefs, health-related QoL, pain, and anxiety and depression	Experimental group: a greater improvement in disability at 3 months follow-up; Catastrophizing, pain, and health-related quality of life improved significantly after the intervention and at follow-up.
[63] Iran	Multicenter assessor-blinded RCT	CNP during COVID- 19 N = 80	PNE: mean age (44.5 ± 6.4), female (55%) PBE: mean age (43.2 ± 7.6), female (47.5%)	Universities, university hospitals, and primary cares	Pain neuroscience education (PNE)-(face-to-face and online)	Pain biomechanics education (PBE)- (face-to-face and online)	Pain and fear of movement	No significant change in pain was observed between the two groups. PNE: significant changes in the fear of movement, intra-group change.
[64] Japan	A single-center, single-arm, open-label feasibility study	chronic neck with shoulder muscle pain/stiffness N = 10	2 males and 8 females, age 43.7 ± 10.3 years (range 29–62 years old)	Hamamatsu University Hospital Translational Research Unit	Light-emitting diodes (LEDs)		Neck and shoulder stiffness, pain, skin temperature, heart rate variability, and baroreceptor reflex sensitivity	Safe and relieve patients’ pain and stiffness.
[68] Germany	Pragmatic randomized trial	Chronic neck pain N = 220	Mean age: 38.9/11.3 years; Female: 67.3%	Universities, gyms, and general practitioners’ offices and local subways	App-based relaxation exercises	Usual care	Pain, pain-related stress, sick-leave days, pain medication intake, and adherence	App did not effectively reduce chronic neck pain or keep the participants engaged in exercising in self-care setting.
[65] Brazil	A randomized Trial	Non-specific CNP N = 90	- Mean age: 40.2/12.3 years - Female: 93.3%	No mention	Osteopathic manipulative treatment (OMT) plus exercise group (EG)	Exercise group (EG)	Pain, DISABILITY, range of motion, fear-avoidance beliefs, and pain self-efficacy	-Outcomes of pain and functionality for patients in both groups were improved at 6 months. - No statistically significant differences in all outcomes were found between OMT/EG and the EG at 3 and 6 months.
[66] Japan	Open-label trial	chronic neck and shoulder discomfort N = 12	mean age: 47 (27–62 years old), 7 females and 5 males	No mention	2-week self-care with gentle mechanical skin stimulation		Pain sensation, discomfort, difficulty in moving; joint range of motions	Improve subjective symptoms and joint ROMs.
[67] Japan	RCT	Chronic neck pain N = 50	- Mean age: 33.1/11.1 years - Female: 62%	A medical school in Tokyo and a medical institution in Kanagawa Prefecture	Acupuncture using press needles	Placebo treatment	Pain, disability, pressure pain threshold	Not significantly reduced pain intensity compared to placebo but reduced the motion-related pain. A direct association was observed between pain severity and the effectiveness of press needles, and the impact of age and computer was indirectly linked to pain severity.
**Qualitative**								
[69] Sweden	A qualitative evaluation	Non-specific Chronic neck and shoulder pain N = 14	All women, mean age 44 years	Primary healthcare center	Feldenkrais groups		Experiences; effects of movement exercises; self-practice - Comparison w/other methods - Opinions	-Positive experiences, especially concerning movement ability and body awareness. -The exercises were difficult to perform as self-training on a daily basis.
[70] UK	A longitudinal qualitative sub-study within the ATLAS trial	Chronic neck pain N = 30		Primary care	Alexander Technique Lessons OR acupuncture sessions	Usual care alone	Perspectives of participants, Self-efficacy, self-care	Positive impact of the interventions on the development of self-care, self-efficacy, and embodiment.

## Appendix C. Description of Study Interventions

**Study (Year)**	**Intervention Components**	**Self-Care Concepts**			**Self-Care Behaviors**
		**Maintenance**	**Monitoring**	**Management**	
[38]	Exercise, physiotherapist’s engagement	*		*	*
[39]	Qigong, exercise therapy, physiotherapists’ breathing techniques, meditation, relaxation, self-performed body massage, engagement, ergonomic instructions, and a pamphlet	*		*	
[40]	Self-SNAGS, along with conventional management	*		*	
[41]	Active wrist acustimulation + standardized neck exercise			*	*
[42]	Therapeutic neck massage, therapists’ Recommendations			*	*
[43]	Qigong, exercise therapy, side-effects report	*		*	*
[44]	Thermotherapy self-treatment, usual medication, and physiotherapy	*		*	
[45]	Self-SNAGs, therapist-administered SNAGs; conventional physiotherapy	*		*	
[46]	Iyengar yoga program			*	
[47]	Yoga course; adherence	*		*	
[48]	Manual therapy, therapeutic patient education, a therapeutic exercise protocol, modify erroneous beliefs about pain and disability, and promote coping strategies and self-efficacy, an information booklet, physiotherapist’s engagement, self-treatment techniques	*		*	*
[49]	Meditation program, adherence, diary	*		*	*
[50]	Acupuncture sessions or Alexander lessons plus usual care	*	*	*	*
[51]	Team learning with self-management strategies; a Pain Diary	*		*	*
[52]	Tai Chi, a training manual, educational units, breathing exercises, relaxation music, neck exercises, proprioceptive exercises, and a daily log	*	*	*	*
[53]	Interactive behavioral modification therapy; progressive neck exercise program; self-report; therapists engagement	*		*	*
[54]	Alexander technique classes; self-observation; Games and partner activities; specialists’ engagement; Electromyography recording		*	*	*
[55]	Alexander lessons plus usual care, acupuncture plus usual care; Self-report questionnaires	*	*	*	*
[56]	Press needle combined with transcutaneous electrical nerve stimulation		*	*	
[57]	Osteopathic manipulative treatment associated with exercise group	*		*	*
[58]	A self-myofascial release therapy			*	
[59]	Guideline-based physical therapy + dry needling	*		*	*
[60]	A general Alexander technique class, an exercise class, and experts’ engagement, Electromyography; Video Game		*	*	*
[61]	Therapeutic exercises alone (TE) or combined (TE + PNE-pain neuroscience education); experts’ engagement			*	*
[62]	An individualized self-management combined with physical therapy intervention	*		*	*
[63]	Pain neuroscience education			*	*
[64]	A single irradiation of high-intensity infrared LED light; testing (temperature, heart rate)		*	*	
[68]	App-based relaxation exercises; notification features, a diary, and questionnaire options	*		*	*
[65]	Osteopathic manipulative treatment (OMT) plus exercise group (EG); screening	*	*	*	*
[66]	Self-care with gentle mechanical skin stimulation; self-observation	*	*	*	
[67]	Acupuncture using press needles			*	*
[69]	Feldenkrais groups; diary; interviews		*	*	*
[70]	Alexander technique lessons OR acupuncture sessions; interviews		*	*	*
*: using one of self-care concepts or self-care behaviors.					

## Appendix D. Self-Care Behaviors by the Middle-Range Theory of Self-Care of Chronic Illness

**Self-Care Component**	**Self-Care Behaviors**	**Study Reference Number**
Self-care maintenance	Stress reduction	[49]
		[51]
		[53]
		[57]
		[62]
		[68]
		[48]
		[52]
		[39]
	Physical activity	[40]
		[47]
		[53]
		[57]
		[59]
		[68]
		[65]
		[48]
		[43]
		[52]
		[39]
		[38]
	Treatment adherence	[44]
		[45]
		[47]
		[50]
		[55]
		[62]
		[68]
		[65]
		[66]
		[48]
		[52]
		[39]
Self-care monitoring	Spatial and body awareness	[50]
		[54]
		[55]
		[60]
		[69]
		[70]
		[52]
	Electromyography recording	[54]
		[56]
	Temperature and heart rate	[64]
	Symptoms observation	[66]
		[65]
Self-care management	Healthcare utilization	[55]
	Musculoskeletal pain management	[40]
		[41]
		[42]
		[44]
		[45]
		[46]
		[47]
		[49]
		[50]
		[51]
		[53]
		[54]
		[55]
		[56]
		[57]
		[58]
		[59]
		[60]
		[61]
		[62]
		[63]
		[64]
		[68]
		[65]
		[66]
		[67]
		[69]
		[70]
		[48]
		[43]
		[52]
		[39]
		[38]
Self-care factors	Self-efficacy	[41]
		[50]
		[53]
		[54]
		[55]
		[57]
		[59]
		[60]
		[61]
		[65]
		[70]
		[48]
	Experience and skill	[42]
		[49]
		[50]
		[59]
		[61]
		[60]
		[62]
		[63]
		[68]
		[67]
		[69]
		[70]
		[43]
		[52]
		[38]
	Job and family life	[51]
		[65]
	Support from others	[51]
		[70]
	Mood states	[57]
